# The Beneficial Effects of Heme Oxygenase 1 and Hydrogen Sulfide Activation in the Management of Neuropathic Pain, Anxiety- and Depressive-like Effects of Paclitaxel in Mice

**DOI:** 10.3390/antiox11010122

**Published:** 2022-01-06

**Authors:** Gerard Roch, Gerard Batallé, Xue Bai, Enric Pouso-Vázquez, Laura Rodríguez, Olga Pol

**Affiliations:** 1 Grup de Neurofarmacologia Molecular, Institut d’Investigació Biomèdica Sant Pau, Hospital de la Santa Creu i Sant Pau, 08041 Barcelona, Spain; gerard.roch@e-campus.uab.cat (G.R.); gerard.batalle@e-campus.uab.cat (G.B.); xue.bai@e-campus.uab.cat (X.B.); enrique.pousovazquez@e-campus.uab.cat (E.P.-V.); laura.rodriguezpe@e-campus.uab.cat (L.R.); 2 Grup de Neurofarmacologia Molecular, Institut de Neurociències, Universitat Autònoma de Barcelona, 08193 Barcelona, Spain

**Keywords:** anxiety, depression, heme oxygenase 1, hydrogen sulfide, chemotherapy-induced peripheral neuropathy, oxidative stress, paclitaxel

## Abstract

Chemotherapy-induced peripheral neuropathy constitutes an unresolved clinical problem that severely decreases the quality of the patient’s life. It is characterized by somatosensory alterations, including chronic pain, and a high risk of suffering mental disorders such as depression and anxiety. Unfortunately, an effective treatment for this neuropathology is yet to be found. We investigated the therapeutic potential of cobalt protoporphyrin IX (CoPP), a heme oxygenase 1 inducer, and morpholin-4-ium 4-methoxyphenyl(morpholino) phosphinodithioate dichloromethane complex (GYY4137), a slow hydrogen sulfide (H_2_S) donor, in a preclinical model of paclitaxel (PTX)-induced peripheral neuropathy (PIPN) in mice. At three weeks after PTX injection, we evaluated the effects of the repetitive administration of 5 mg/kg of CoPP and 35 mg/kg of GYY4137 on PTX-induced nociceptive symptoms (mechanical and cold allodynia) and on the associated emotional disturbances (anxiety- and depressive-like behaviors). We also studied the mechanisms that could mediate their therapeutic properties by evaluating the expression of key proteins implicated in the development of nociception, oxidative stress, microglial activation, and apoptosis in prefrontal cortex (PFC) and dorsal root ganglia (DRG) of mice with PIPN. Results demonstrate that CoPP and GYY4137 treatments inhibited both the nociceptive symptomatology and the derived emotional alterations. These actions were mainly mediated through potentiation of antioxidant responses and inhibiting oxidative stress in the DRG and/or PFC of mice with PIPN. Both treatments normalized some plasticity changes and apoptotic reactions, and GYY4137 blocked microglial activation induced by PTX in PFC. In conclusion, this study proposes CoPP and GYY4137 as good candidates for treating neuropathic pain, anxiety- and depressive-like effects of PTX.

## 1. Introduction

During the last several decades, the survival rates for most cancers have been increasing, an improvement that can be partially attributed to new and improved treatments [1]. Unfortunately, the same tendency is not observable for many of the secondary effects of oncotherapies, which remain unresolved and affect a high number of patients. Chemotherapy-induced peripheral neuropathy (CIPN) is a side effect that has significant impacts, both on cancer survival and on the patient’s quality of life; it is a crucial limiting factor that can lead to a change or reduction in the therapy or, in severe cases, to its total cessation [1]. 

The usual CIPN symptomatology includes multiple somatosensory alterations, such as allodynia, hyperalgesia, and spontaneous pain such as burning spams [2]. Remarkably, different studies have also concluded that, if CIPN persists over time, this symptomatology greatly increases the risk of developing severe emotional disorders such as anxiety, depression, and insomnia. In some cases, these tendencies are maintained long after treatment cessation, reinforcing the conception of CIPN as a chronic disease [3]. Unfortunately, there are currently no effective pharmacological therapies for CIPN, and the efficacy of the existing therapies is only moderate [4].

In the last decade, innovative pharmacological approaches have been tested in animal trials with positive analgesic results, e.g., the administration of reactive oxygen species (ROS) scavengers [5,6], antioxidants such as oltipraz [7], or anti-inflammatory substances [8]. However, very few studies have investigated new possible effective treatments for the emotional disorders associated with CINP. Considering that mental illnesses are hard to treat once established and that they often persist after the original cause has been resolved, it is crucial to assess the possible anxiolytic and antidepressant effects of the analgesics to improve cancer patients’ mental health.

Carbon monoxide (CO) is a gaseous neurotransmitter with relevant implications in pain processing [9,10]. Accordingly, CO-releasing molecules and heme oxygenase 1 (HO-1) enzyme inducers have been demonstrated to be effective analgesic options in inflammatory and neuropathic pain models [11,12,13]. Additionally, different studies indicate that these substances have anxiolytic effects [10,11,12,13,14].

Hydrogen sulfide (H_2_S), another gaseous neurotransmitter, also plays an important role in nociception [15]. Numerous studies have reported the painkilling actions of this neurotransmitter when administering slow H_2_S-releasing agents such as isothiocyanates or morpholin-4-ium 4-methoxyphenyl(morpholino) phosphinodithioate dichloromethane complex (GYY4137) in animals with osteoarthritic or nerve-injury-induced neuropathic pain [16,17,18]. These treatments also improved the affective behaviors accompanying chronic osteoarthritic pain and neuropathic pain generated by nerve injury or associated with diabetes [17,19,20]. 

Paclitaxel (PTX) is a taxane chemotherapeutic agent widely used for the treatment of cancer, including ovarian, breast, lung, and pancreatic cancer [21]. It has one of the highest CIPN incidence rates and the CIPN may persist for a long time, and it is accompanied by multiple affective disorders that interfere with positive outcomes [22,23]. In this study, we used a preclinical model of PTX-induced peripheral neuropathy (PIPN) developed by [24], which reproduces the somatosensory clinical features such as the bilateral mechanical and cold allodynia, as well as the anxiodepressive-like behaviors present in humans [22]. In this model, we evaluated the effects induced by two well-recognized antioxidant compounds: cobalt protoporphyrin IX (CoPP), an inducer of the antioxidant enzyme HO-1, and GYY4137, a slow-release H_2_S donor, which also exhibits important antioxidant properties in several diseases.

The mitogen-activated protein kinase (MAPK) and phosphatidylinositol 3-kinase (PI3K)/protein kinase B (Akt) signaling pathways are activated after PTX injection and are actively involved in the establishment of PIPN [25,26]. In this study, we evaluated the effects of CoPP and GYY4137 on the protein levels of p-P38, p-ERK1/2, PI3K, and p-Akt in the prefrontal cortex (PFC) and dorsal root ganglia (DRG), two specific areas sited in the central (CNS) and peripheral (PNS) nervous system implicated in the modulation of nociception and emotional disorders [27,28]. 

Oxidative stress also plays a crucial role in the development of CIPN [28]. Previous studies have shown an upregulation of several oxidative-stress markers and/or the downregulation of the nuclear factor erythroid-2-related factor 2 (Nrf2), HO-1, NAD(P)H quinone dehydrogenase 1 (NQO1) and superoxide dismutase 1 (SOD-1) axis in animals with CINP [28,29]. Therefore, the activation of Nrf2 and HO-1 improves CIPN symptomatology [11,12]. We analyzed the effects of CoPP and GYY4137 on the protein levels of the oxidative biomarkers 4-hydroxynonenal (4-HNE) and malondialdehyde (MDA) and of the antioxidant enzymes HO-1, NQO1, and SOD-1 in the PFC and DRG of PTX-injected mice.

Microglia are also implicated in the establishment of CIPN [30] and play a crucial role in the development of emotional disorders accompanying chronic pain [31]. The possible contribution of microglia to the effects of both treatments was also assessed.

Finally, because the deregulation in calcium homeostasis and in the antioxidant response elements induced by PTX provoke apoptotic responses [32], the BAX expression in the PFC and DRG of PTX-injected mice treated with CoPP or GYY4137 was also analyzed.

## 2. Materials and Methods

### 2.1. Animals

Male 5-6-week-old C57BL/6 mice (21–25 g), acquired from Envigo Laboratories (Barcelona, Spain), were used for the experiments. They were kept within a plastic cage under controlled environmental conditions (a temperature of 22 °C, a humidity of 66%, and a 12 h dark/light cycle), and an ad libitum food and water supply was provided. A 7-day acclimatation period to the housing conditions took place before starting the experiments, which were conducted between 9:00 a.m. and 5:00 p.m. Animals were executed in strict accordance with the guidelines of the European Commission’s directive (2010/63/EC) and the Spanish Law (RD 53/2013) regulating animal research, and the procedure was approved by the local Committee of Animal Use and Care of the Autonomous University of Barcelona (ethical code is 9863). All efforts were made to minimize both the number of animals used and their suffering.

### 2.2. PTX-Derived Peripheral Neuropathy Induction

**Figure 1:**
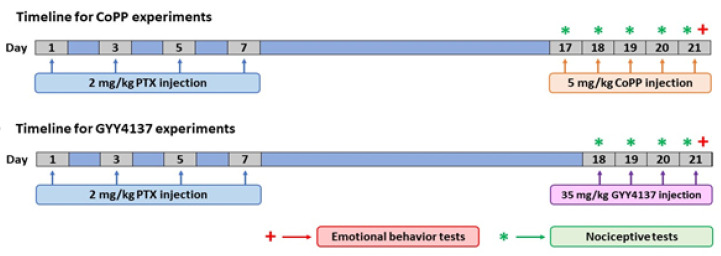
Schematic illustration of the design of the experiment, performed to determine whether the repetitive administration of CoPP (5 mg/kg) or GYY4137 (35 mg/kg) intraperitoneally injected over 5 and 4 consecutive days, respectively, can reverse the nociceptive responses caused by PTX and the associated emotional disorders. PTX: paclitaxel.

Painful neuropathy was induced with PTX (Tocris Bioscience, Bristol, UK) intraperitoneally (i.p.) injected at 2 mg/kg, every other day, for four consecutive days (Figure 1), as per a previous study [24]. PTX was dissolved in Cremophor EL (Sigma-Aldrich, St. Louis, MO, USA)/ethanol/saline (SS, 0.9% NaCl) in a mixture of 1:1:18. The control group received an equal volume of vehicle solution in the same schedule.

### 2.3. Nociceptive Tests

Mechanical allodynia was evaluated by measuring the hind paw withdrawal response after the stimulation with the von Frey (VF) filaments of different bending forces (0.008–3.5 g). Animals were placed inside individual methacrylate cylinders (20 cm high × 9 cm in diameter; Servei Estació, Barcelona, Spain) with a grid bottom through which the filaments (North Coast Medical, Inc., San Jose, CA, USA) were perpendicularly applied in the central area of the hind paws when mice were resting by using the up–down paradigm. The VF hairs were pressed for 2–3 s, starting with a filament of 0.4 g, and the stiffness of the following hair to be used was decided based on the elicited response. The threshold of the response was calculated using an Excel program (Microsoft Iberia SRL, Barcelona, Spain) that included curve fitting of the data. A fast withdrawal, licking, or shaking of the paw was registered as a positive response.

Cold allodynia was assessed by using a cold plate (CP) analgesiometer (Ugo Basile, Italy). The plate temperature was set at 4 ± 0.5 °C, and the number of elevations for each hind paw was registered for 5 min.

In all tests, animals were habituated to the environment for 1 h before the experiment. Both ipsilateral and contralateral paws were tested.

### 2.4. Emotional Behavior Tests

The anxiety-like behavior was assessed by utilizing the elevated plus maze (EPM) [33] and the open file (OF) tests [34].

The EPM apparatus used in this test had 4 arms, each of them 5 cm wide and 35 cm long. Two of them were closed by 15 cm high walls, while the other two were open. The maze was suspended 45 cm above the floor. Mice were placed in the center of the structure, always facing the same open arm, and allowed to explore for 5 min. Their movements were recorded by a digital camera. We registered the number of entries into the open and closed arms, and the percentage of time spent in the open arms. 

In the OF test, animals were placed in the center of a 44 cm × 44 cm box enclosed by grey walls that were 30 cm high. They were allowed to freely explore this space for 5 min, and their behavior during this time was recorded by a digital camera. The number of squares crossed and entries into the central area and the amount of time spent in this central area were considered.

The evaluation of the depressive-like behaviors was performed by using the tail suspension test (TST) and the forced swimming test (FST), by which the duration of immobility of the animals was quantified according to the methods described in [35,36].

In the TST, animals isolated acoustically and visually were suspended at 35 cm from the floor by applying adhesive tape to the tip of the tail and attaching it to an elevated surface. Their movements were recorded for 8 min with a digital camera. The first 2 min were considered habituation time and were not analyzed. The amount of time spent in total immobility during the following 6 min was registered. 

In the FST, animals were placed in methacrylate cylinders (25 cm high × 10 cm in diameter; Servei Estació, Barcelona, Spain) filled with tempered water (24 ± 2 °C) up to a 10 cm depth. Mice were then carefully placed in the water and left in this environment for 6 min. Their activity was recorded by a digital camera. The first 2 min were considered habituation time and were not analyzed. The amount of time spent in immobility during the following 4 min was registered.

In both tests, mice were considered immobile when they remained completely still.

### 2.5. Western Blot Analysis

Mice were euthanized by cervical dislocation at day 21 post-injection (PTX or vehicle). Tissues from the PFC and DRG were immediately bilaterally extracted, frozen in dry ice, and stored at −80 °C until use. Cell disruption was done in an ice-cold lysis RIPA buffer (Sigma-Aldrich) plus a 0.5% protease inhibitor cocktail (Sigma-Aldrich) and a 1% phosphatase inhibitor cocktail (Sigma-Aldrich). A two-step sonication process (20 kHz, 10 s cooldown in ice) was used, leaving the samples at 4 °C for 1 h in the lysis buffer between the steps. Samples were then centrifuged for 20 min at 9500 rpm and at 4 °C. The supernatant was subsequently collected, and its protein concentration was quantified through a BCA assay by a plate reader (BioTek Synergy HT, Winooski, VT, USA) and stored at −80 °C until use. For the electrophoresis, 60 µg of total protein were mixed with a 4x Laemmli loading buffer and loaded into 4% stacking/12% separating sodium dodecyl sulfate polyacrylamide gels. Thereafter, proteins were transferred onto a polyvinylidene fluoride membrane by electrophoresis (120 min) and blocked for 75 min with one of the following solutions: phosphate-buffered saline (PBS) + 5% nonfat dry milk, PBS with Tween 20 (PBST) + 5% bovine serum albumin (BSA), or Tris-buffered saline with Tween 20 (TBST) + 5% nonfat dry milk or 5% BSA.

Membranes were then incubated overnight at 4 °C with rabbit primary antibodies against the following: p-P38 (1:200), P38 (1:250), p-ERK 1/2 (1:250), ERK 1/2 (1:250), p-Akt (1:200), Akt (1:200), and BAX (1:150), which were obtained from Cell Signaling Technology (Danvers, MA, United States); 4-HNE (1:150), MDA (1:150), PI3K (1:150), and HO-1 (1:150), which were bought at Abcam (Cambridge, United Kingdom); CD11b/c (1:200) and SOD-1 (1:150), which were acquired from Novus Biologic (Littleton, CO, USA); and NQO1 (1:250) and β-actin (1:5000), which were obtained from Merck (Billerica, MA, USA). β-actin was used as the loading control. Afterwards, blots were incubated at room temperature for 1 h with a horseradish peroxidase-conjugated anti-rabbit or anti-mouse secondary antibodies (GE Healthcare, Little Chalfont, United Kingdom). Finally, chemiluminescence reagents (ECL kit; GE Healthcare, Little Chalfont, United Kingdom) were added, and photon emission was scanned using the Chemidoc MP imaging system (Bio Rad, Hercules, CA, USA). Band density was determined and compared with the Image-J software (version 1.34s, National Institutes of Health, Bethesda, MD, USA).

### 2.6. Experimental Procedures

We evaluated the analgesic, anxiolytic, and/or antidepressant properties of CoPP and GYY4137 in PTX-injected mice. To this end, mice were injected daily for five consecutive days with 5 mg/kg CoPP or vehicle, at days 17, 18, 19, 20, and 21 after PTX injection. For GYY4137, mice received four daily injections of 35 mg/kg of this drug or vehicle at days 18, 19, 20, and 21 after PTX injection, as depicted in Figure 1.

For both treatments, nociceptive tests (VF and CP) were carried out on the same days of drug injection, while the emotional behavior tests (EPM, OF, TST, and FST) were performed at the end of treatment, on day 21 after PTX injection. For each drug, nociceptive and emotional behaviors were tested in different groups of mice. All animals were sacrificed at day 21 after PTX injection (*n* = 6–8 animals per group).

All these experiments were performed by experimenters blinded to the experimental conditions and the animals receiving the experimental treatment or vehicle were injected and tested at the same day under the same conditions.

### 2.7. Drugs

CoPP, obtained from Frontier Scientific (Livchem GmbH & Co., Frankfurt, Germany), dissolved in dimethyl sulfoxide (1% in SS) was i.p. administered at 5 mg/kg daily for five consecutive days, 3 h before testing, in accordance with [14]. GYY4137, acquired from Sigma-Aldrich (St. Louis, MO, USA), dissolved in SS was i.p. administered at 35 mg/kg daily for four consecutive days, 1 h before testing, in accordance with [20]. Both compounds were injected in a final volume of 10 mL/kg. All drugs were freshly prepared before use. For each group treated with a drug, the respective control group received the same volume of the corresponding vehicle.

### 2.8. Statistical Analyses

The statistical studies were performed with Prism 8.0 (Graphpad, La Jolla, CA, USA). The results are expressed as the mean values ± standard error of the mean (SEM). The two-way repeated measures ANOVA, with treatment and time as the variation factors, the one-way ANOVA, and the Student–Newman–Keuls (SNK) test were used to assess the effects of CoPP and GYY4137 administration on PTX-induced nociception. The effects of both treatments on the emotional behaviors were analyzed by using the one-way ANOVA with treatment as the variation factor, followed by the corresponding SNK post-hoc test. The differences in protein expression were also evaluated using the one-way ANOVA followed by the SNK test. A value of *p* < 0.05 was considered significant.

## 3. Results

### 3.1. Treatment with CoPP Inhibited the Mechanical and Cold Allodynia Caused by PTX

In both paws, two-way repeated-measures ANOVA showed the significant effects of the treatment, time, and their interaction (*p* < 0.001) for the mechanical and thermal allodynia. 

**Figure 2:**
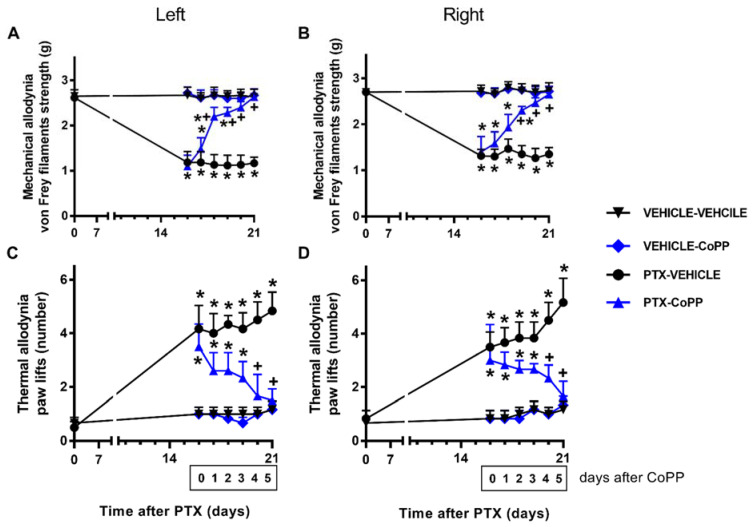
Effects of CoPP on PTX-induced mechanical and cold allodynia. Mechanical (**A**: left paws; **B**: right paws) and cold (**C**: left paws; **D**: right paws) antiallodynic effects produced by the repeated administration of 5 mg/kg of CoPP or vehicle for five days from days 17 to 21 after PTX injection. In all panels, for each day and treatment evaluated, * indicates significant differences vs. the vehicle–vehicle-treated animals and + vs. PTX–vehicle-treated animals (*p* < 0.05, one-way ANOVA followed by the SNK test). Results are shown as mean values ± SEM; *n* = 6 animals per experimental group.

Concerning the mechanical allodynia, the administration of CoPP progressively reduced the mechanical allodynia induced by PTX from days 1 to 3 of treatment and completely inhibited it at day 5 of treatment in both hind paws (*p* < 0.001; one way ANOVA and SNK test; Figure 2A,B). Similar results were obtained for cold allodynia, as CoPP treatment significantly decreased the number of times that mice elevated both hind paws in the cold plate from days 1 to 3 of treatment, until complete reversal was achieved at day 5 of treatment (*p* < 0.014, one-way ANOVA followed by SNK tests; Figure 2C,D). In both tests and paws, CoPP did not produce any effect in the vehicle-injected mice.

### 3.2. CoPP Treatment Prevented the Emotional Behavioral Alterations Associated with PIPN

**Figure 3:**
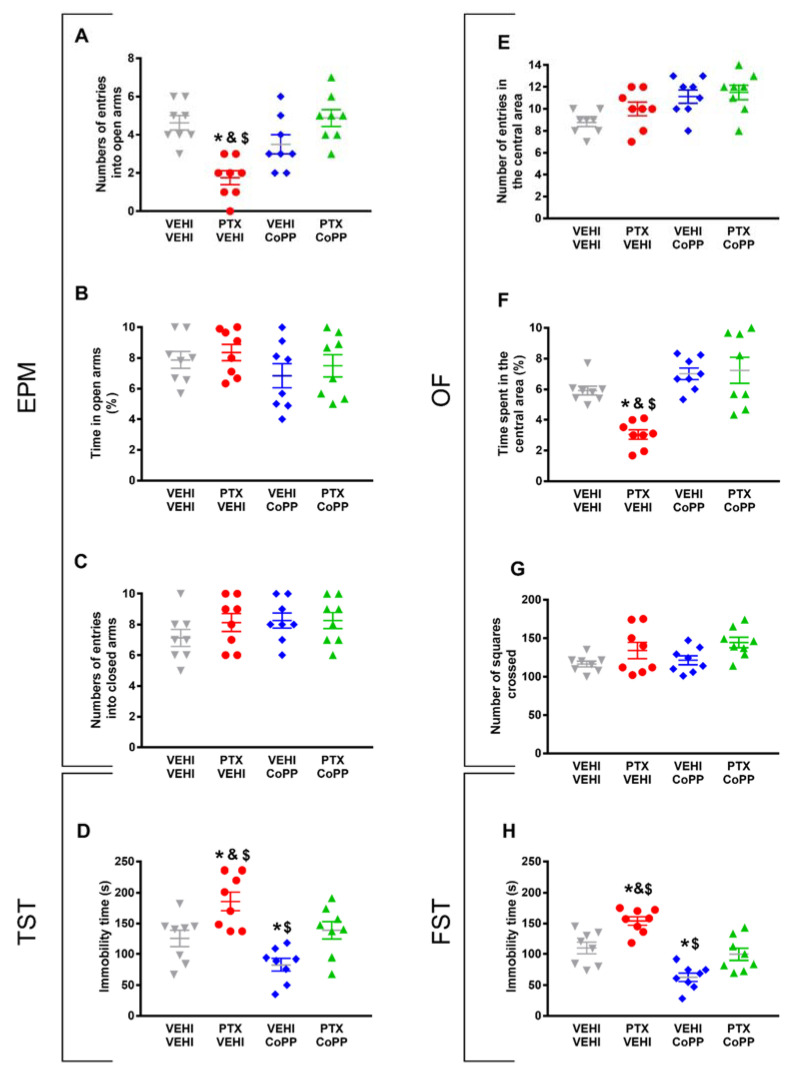
Effects of treatment with CoPP on the emotional behavioral disorders associated with PTX-induced neuropathic pain. Effects of the repetitive administration of 5 mg/kg of CoPP or vehicle from days 17 to 21 after PTX or vehicle (VEHI) injection on the anxiety- (EPM and OF tests) and depressive-like behaviors (TST and FST) associated with CIPN. In the EPM test, the number of times that animals entered the open arms (**A**), the percentage of total time spent in open arms (**B**), and the number of entrances into the closed arms (**C**) are represented. In the OF test, the number of entries into the central area (**E**), the percentage of time spent within the central area (**F**), and the total number of squares crossed (**G**) are shown. Figures (**D**,**H**) display the time the animals spent in immobility (s) in the TST (**D**) and FST (**H**). In all panels, * indicates significant differences vs. the vehicle–vehicle-treated animals; &, vs. the vehicle–CoPP-treated animals; and $, vs. the animals treated with PTX plus CoPP (*p* < 0.05, one-way ANOVA followed by the SNK test). Data are expressed as mean values ± SEM; *n* = 8 animals per experimental group.

Regarding the anxiety-like behaviors, CoPP reversed both the decreased number of entries into the EPM’s open arms (*p* < 0.021, one-way ANOVA followed by the SNK test as compared with the vehicle–vehicle-treated mice; Figure 3A) and the decreased time spent in the central area of the OF in PTX-injected animals (*p* < 0.035, one-way ANOVA followed by the SNK test as compared with the vehicle–vehicle-treated mice; Figure 3F).

The percentage of time spent in the open arms (Figure 3B) and the number of entries into the closed arms of the EPM (Figure 3C), as well as the number of entries in the central area (Figure 3E) and the number of squares crossed in the OF (Figure 3G) did not show significant differences among the groups.

The results regarding the depressive-like behaviors associated with PTX-induced neuropathic pain also revealed that CoPP normalized the increased immobility time observed in PTX–vehicle-treated animals in both the TST (*p* < 0.001, one-way ANOVA followed by the SNK test; Figure 3D) and FST (*p* < 0.001; one-way ANOVA followed by the SNK test; Figure 3H). This treatment also decreased the immobility time in the vehicle-injected animals in both tests (*p* < 0.001; one-way ANOVA followed by the SNK test, as compared with their corresponding vehicle–vehicle-treated animals) (Figure 3D,H), thus revealing its antidepressant effects in basal conditions.

Overall, our results indicated that the administration of 5 mg/kg of CoPP for five consecutive days reverted the mechanical and cold allodynia induced by PTX and normalized the accompanying anxiety- and depressive-like behaviors.

### 3.3. GYY4137 Inhibited the Mechanical and Cold Allodynia Induced by PTX

**Figure 4:**
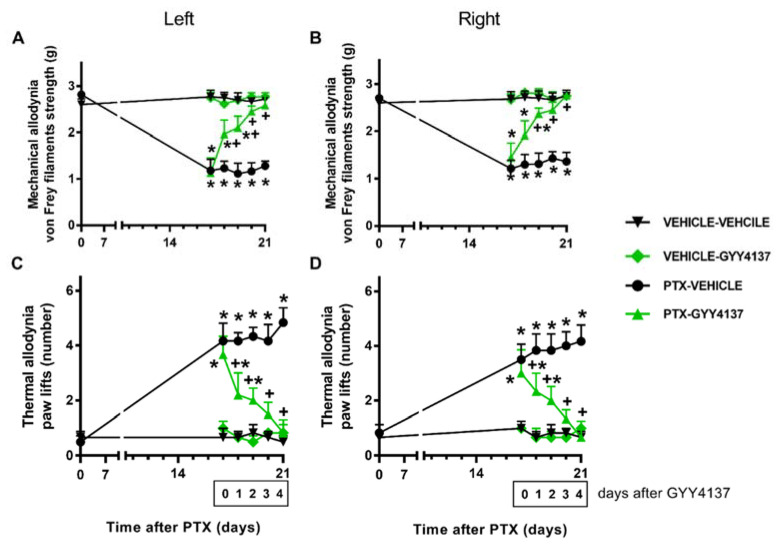
Effects of GYY4137 on PTX-induced nociception. Mechanical (**A**: left paws; **B**: right paws) and cold (**C**: left paws; **D**: right paws) antiallodynic effects produced by the repeated administration of 35 mg/kg of GYY4137 or vehicle for four days from days 18 to 21 after PTX injection. In all graphs, for each day and treatment evaluated, * indicates significant differences vs. the vehicle–vehicle-treated animals, and +, vs. the PTX–vehicle-treated animals (*p* < 0.05, one-way ANOVA followed by the SNK test). Results are shown as mean values ± SEM; *n* = 6 animals per experimental group.

Concerning PTX-induced mechanical and cold allodynia, two-way repeated-measures ANOVA revealed the significant effects of treatment, time, and their interaction (*p* < 0.001). Our data further demonstrate that GYY4137 administration progressively reduced the mechanical allodynia from days 1 to 2 of treatment in the left and right hind paws (*p* < 0.001; one-way ANOVA and the SNK test vs. their corresponding vehicle–vehicle-treated animals) (Figure 4A,B). On the fourth day of treatment, complete inhibition was achieved in both hind paws.

Similar results were obtained for cold allodynia, where GYY4137 treatment gradually diminished the number of both hind paw elevations in the CP after 1 and 2 days of treatment (*p* < 0.001; one-way ANOVA and the SNK test vs. their corresponding vehicle–vehicle-treated group). Total reversal was accomplished in both hind paws at day 4 of treatment (Figure 4C,D). GYY4137 did not produce any significant effects in the vehicle-injected animals in any of these tests.

### 3.4. GYY4137 Administration Relieved PIPN-Derived Affective Disorders

**Figure 5:**
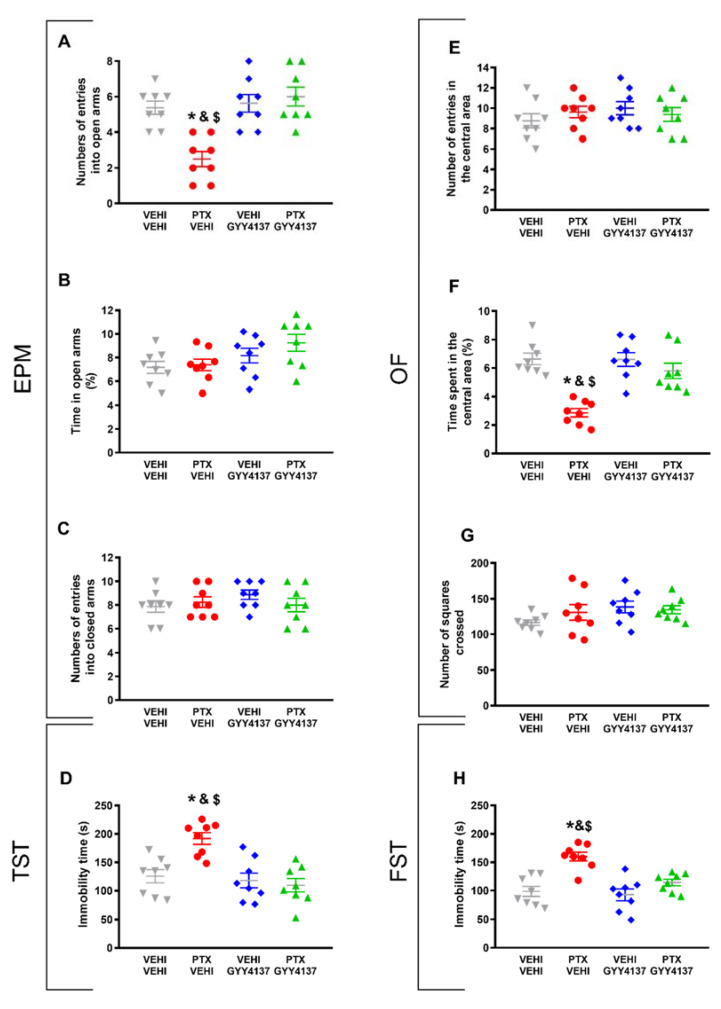
Effects of GYY4137 treatment on the emotional disorders accompanying PTX-induced neuropathic pain. Effects of the repetitive administration of 35 mg/kg of GYY4137 or vehicle from days 18 to 21 after PTX or vehicle (VEHI) injection on the anxiety- (EPM and OF tests) and depressive-like behaviors (TST and FST) associated with CIPN. In the EPM test, the number of times that animals entered into the open arms (**A**), the percentage of total time spent in open arms (**B**), and the number of entrances into the closed arms (**C**) are represented. In the OF test, graphs show the number of entries into the central area (**E**), the percentage of time spent within the central area (**F**), and the total number of squares crossed (**G**). Figures (**D**,**H**) display the time spent in immobility (s) in the TST (**D**) and the FST (**H**). In all panels, * indicates significant differences vs. the vehicle–vehicle-treated mice; &, vs. the vehicle–GYY4137-treated animals; and $, vs. the PTX–GYY4137-treated animals (*p* < 0.05, one-way ANOVA followed by the SNK post hoc test). Data are expressed as mean values ± SEM; *n* = 8 animals per experimental group.

In reference to the anxiety-like behaviors accompanying PTX-induced neuropathic pain, our results demonstrate that GYY4137 reversed both the decreased number of entries into the open arms in the EPM (*p* < 0.016; one-way ANOVA followed by the SNK test in comparison with vehicle–vehicle-treated animals; Figure 5A) and the diminished time spent in the central area of the OF observed in the PTX-injected mice (*p* < 0.032; one-way ANOVA followed by the SNK test as compared with the vehicle–vehicle-treated animals; Figure 5F). No significant differences in the percentage of time spent in the open arms (Figure 5B) or the number of entries into the closed arms of the EPM (Figure 5C), or in the number of entries in the central area (Figure 5E) or the number of squares crossed in the OF (Figure 5G) were detected among any of the groups. 

In the depressive-like behavior tests, GYY4137 stabilized the high time spent in immobility observed in PTX-injected mice, both in the TST (*p* < 0.005; one-way ANOVA followed by the SNK test comparing with vehicle–vehicle group; Figure 5D) and in the FST (*p* < 0.002; one-way ANOVA followed by SNK vs. the vehicle–vehicle-treated mice; Figure 5H). 

Taken together, these results indicate that the administration of 35 mg/kg of GYY4137 for four consecutive days reversed PTX-provoked mechanical and cold allodynia, as well as the associated anxiety- and depressive-like behaviors.

### 3.5. Effects of CoPP and GYY4137 on the Expression of MAPK and PI3K/p-Akt in PFC and DRG of PTX-Injected Mice

**Figure 6:**
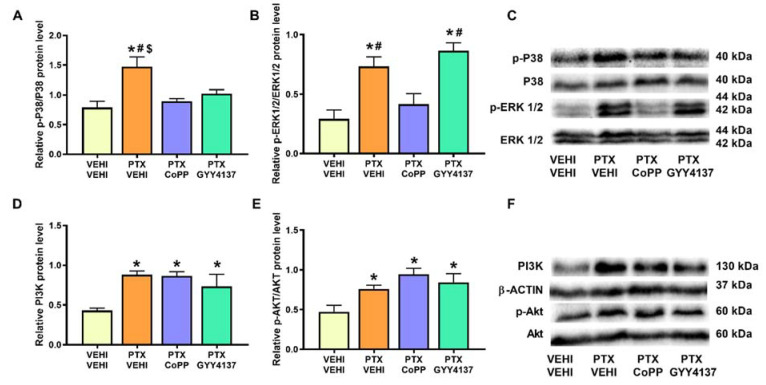
Effects of CoPP and GYY4137 on the expression of p-P38, p-ERK 1/2, PI3K, and p-Akt in the PFC of PTX-injected mice. The graphs represent the protein levels of p-P38/P38 (**A**), p-ERK 1/2/ERK 1/2 (**B**), PI3K (**D**), and p-Akt/Akt (**E**) in the PFC of PTX-injected mice treated with CoPP or GYY4137. The control group treated with VEHI–VEHI is also shown. Representative blots for p-P38, P38, p-ERK 1/2, and ERK 1/2 (**C**) and for PI3K, β-actin, p-Akt, and Akt (**F**) are displayed. In all panels, * indicates significant differences vs. the VEHI–VEHI-treated mice; #, vs. the PTX–CoPP-treated mice; and $, vs. the PTX–GYY4137 treated animals (*p* < 0.05, one-way ANOVA followed by the SNK post hoc test). Data are expressed as mean values ± SEM; *n* = 3 samples per group.

In PFC, PTX induced the phosphorylation of P38 (*p* < 0.010, one-way ANOVA followed by the SNK test in comparison to the vehicle–vehicle group; Figure 6A) and ERK 1/2 (*p* < 0.001, one-way ANOVA followed by the SNK test in contrast to the vehicle–vehicle group; Figure 6B). PTX also increased the PI3K (*p* < 0.016; one-way ANOVA followed by the SNK test compared with the vehicle–vehicle-treated mice; Figure 6D) and p-Akt levels (*p* < 0.0173, one-way ANOVA followed by SNK vs. the vehicle–vehicle group; Figure 6E). Our results show that both the CoPP and GYY4137 treatments normalized p-P38 levels and CoPP reversed the p-ERK 1/2 overexpression. Neither CoPP nor GYY4137 prevented the PTX-induced PI3K and p-Akt upregulation.

**Figure 7:**
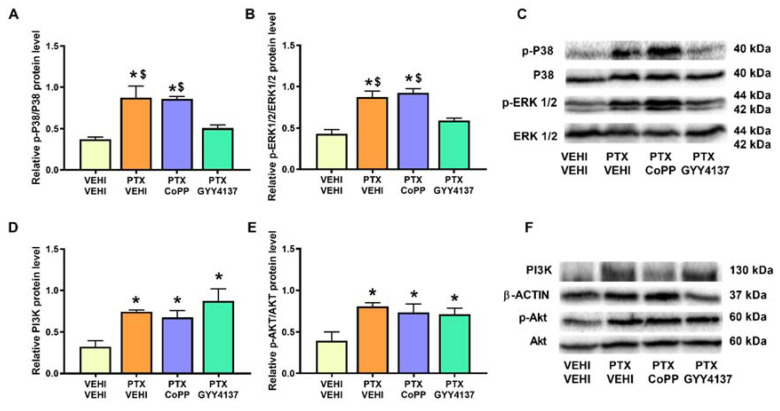
Effects of CoPP and GYY4137 on the expression of p-P38, p-ERK 1/2, PI3K, and p-Akt in the DRG of PTX-injected mice. The graphs represent the protein levels of p-P38/P38 (**A**), p-ERK 1/2/ERK 1/2 (**B**), PI3K (**D**), and p-Akt/Akt (**E**) in the DRG of PTX-injected mice treated with CoPP or GYY4137. The control group treated with VEHI–VEHI is also shown. Representative blots for p-P38, P38, p-ERK 1/2, and ERK 1/2 (**C**) and for PI3K, β-actin, p-Akt, and Akt (**F**) are displayed. In all panels, * indicates significant differences vs. the VEHI–VEHI-treated mice, and $, vs. PTX-injected mice treated with GYY4137 (*p* < 0.05, one-way ANOVA followed by the SNK post hoc test). Data are expressed as mean values ± SEM; *n* = 3 samples per group.

Analogously to the results obtained in the PFC, significant increases in the phosphorylated forms of P38 (*p* < 0.005; one-way ANOVA followed by the SNK test in comparison to the vehicle–vehicle group; Figure 7A), ERK 1/2 (*p* < 0.001; one-way ANOVA followed by the SNK test vs. the vehicle–vehicle mice; Figure 7B), and Akt (*p* < 0.011; one-way ANOVA followed by the SNK test in contrast to the vehicle–vehicle-treated mice; Figure 7E) were detected in the DRG. PI3K expression was also increased in the PTX-injected mice (*p* < 0.0114; one-way ANOVA followed by the SNK test as compared to vehicle–vehicle-treated animals; Figure 7D). In this tissue, only GYY4137 normalized the upregulation of p-P38 and p-ERK 1/2. Neither CoPP nor GYY4137 altered the expression of PI3K or p-Akt.

**Figure 8:**
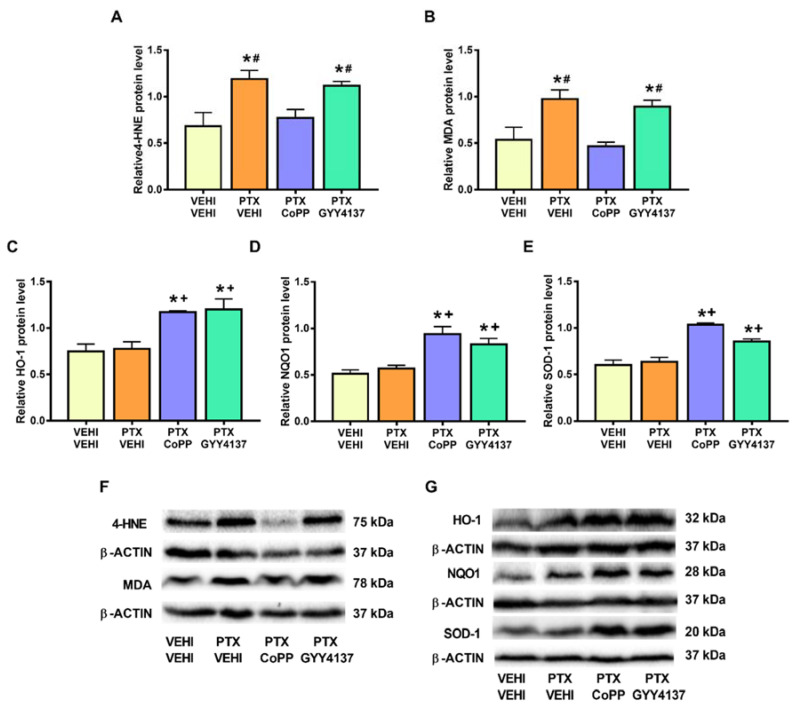
Effects of CoPP and GYY4137 on the levels of 4-HNE, MDA, HO-1, NQO1, and SOD1 in the PFC of PTX-injected mice. Graphs represent the levels of 4-HNE (**A**), MDA (**B**), HO-1 (**C**), NQO1 (**D**), and SOD-1 (**E**) in the PFC of PTX-injected animals treated with CoPP or GYY4137. The control group treated with VEHI–VEHI is also represented. Representative blots for 4-HNE and MDA (**F**) and for HO-1, NQO1, and SOD1 (**G**) are shown. All proteins are represented relative to β-actin levels. In all panels, * indicates significant differences vs. the VEHI–VEHI-treated mice; +, vs. animals treated with PTX–VEHI; and #, vs. the PTX–CoPP treated mice (*p* < 0.05, one-way ANOVA followed by the SNK post hoc test). Data are expressed as mean values ± SEM; *n* = 3 samples per group.

Regarding oxidative stress, PTX significantly increased the 4-HNE (*p* < 0.008; one-way ANOVA followed by the SNK test as compared with the vehicle–vehicle group; Figure 8A) and MDA levels (*p* < 0.002; one-way ANOVA followed by the SNK test as compared with the vehicle–vehicle group; Figure 8B), which were normalized by CoPP treatment. Treatment with CoPP and GYY4137 also increased the expression of HO-1 (*p* < 0.0016; one-way ANOVA followed by the SNK test as compared with the vehicle–vehicle-treated animals; Figure 8C), NQO1 (*p* < 0.001; one-way ANOVA followed by the SNK test as compared with the vehicle–vehicle group; Figure 8D), and SOD-1 (*p* < 0.001; one-way ANOVA followed by the SNK test in comparison to the vehicle–vehicle group; Figure 8E).

**Figure 9:**
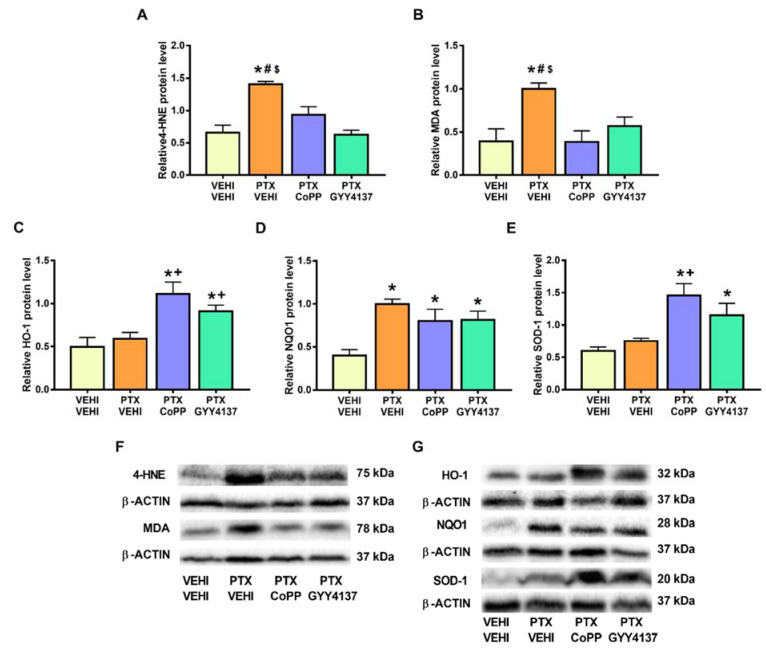
Effects of CoPP and GYY4137 on the levels of 4-HNE, MDA, HO-1, NQO1, and SOD1 in the DRG of PTX-injected mice. Graphs represent the levels of 4-HNE (**A**), MDA (**B**), HO-1 (**C**), NQO1 (**D**), and SOD-1 (**E**) in the DRG of PTX-injected animals treated with CoPP or GYY4137. The control group treated with VEHI–VEHI is also represented. Representative blots for 4-HNE and MDA (**F**) and for HO-1, NQO1, and SOD1 (**G**) are shown. All proteins are represented relative to β-actin levels. In all panels, * indicates significant differences vs. the VEHI–VEHI-treated mice; +, vs. animals treated with PTX–VEHI; #, vs. the PTX–CoPP-treated mice; and $, vs. mice treated with PTX plus GYY4137 (*p* < 0.05, one-way ANOVA followed by the SNK post hoc test). Data are expressed as mean values ± SEM; *n* = 3 samples per group.

In the DRG, PTX increased the 4-HNE and MDA levels (*p* < 0.002; one-way ANOVA followed by the SNK test as compared with their respective vehicle–vehicle group; Figure 9A,B) and those of NQO1 (*p* < 0.001; one-way ANOVA followed by the SNK test as compared to the vehicle–vehicle group; Figure 9D). Both the CoPP and GYY4137 treatments normalized the 4-HNE and MDA levels and maintained the high levels of NQO1 induced by PTX. Both treatments also increased the expression of HO-1 (*p* < 0.005; one-way ANOVA followed by the SNK test in comparison to mice treated with vehicle–vehicle and PTX-vehicle; Figure 9C) and SOD-1 (*p* < 0.001; one-way ANOVA followed by the SNK test in comparison to mice treated with vehicle–vehicle and/or PTX-vehicle; Figure 9E).

**Figure 10:**
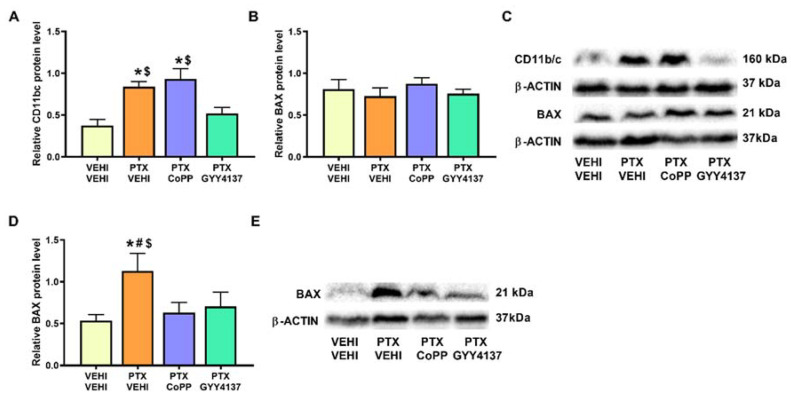
Effects of CoPP and GYY4137 treatments on the expression of CD11b/c in the PFC and BAX in the PFC and DRG of PTX-injected mice. Graphs represent the expression of CD11b/c in PFC (**A**), and BAX in the PFC (**B**) and DRG (**D**) of PTX-injected mice treated with CoPP or GYY4137. Animals treated with VEHI–VEHI were used as controls. Representative blots for CD11b/c and BAX (**C**) in the PFC and for BAX in the DRG (**E**) are shown. All proteins are represented relative to β-actin levels. In all panels, * indicates significant differences vs. the VEHI–VEHI-treated animals; #, vs. the PTX–CoPP-treated animals; and $, vs. PTX-injected mice treated with GYY4137 (*p* < 0.05, one-way ANOVA followed by the SNK post hoc test). Data are expressed as mean values ± SEM; *n* = 3 samples per group.

Regarding microglial activation, a significant increase in CD11b/c was observed in the PFC of PTX-injected mice (*p* < 0.007; one-way ANOVA followed by the SNK test as comparing with vehicle–vehicle group; Figure 10A). This increase was normalized by GYY4137 but not by CoPP treatment. The results for the apoptotic marker BAX revealed that, although no significant differences in its expression were observed in the PFC of PTX-injected mice (Figure 10B), an increase in its expression was detected in the DRG (*p* < 0.029; one-way ANOVA followed by the SNK test vs. the vehicle–vehicle-treated animals; Figure 10D). Both treatments reversed the BAX overexpression induced by PTX in the DRG.

## 4. Discussion

This study proved the analgesic, anxiolytic, and antidepressant properties of an HO-1 inducer (CoPP) and slow H_2_S releaser (GYY4137) in a murine model of PIPN and furthermore revealed their effects in the CNS and PNS. 

PIPN causes great suffering to cancer patients, and no effective treatments for this type of neuropathy and its associated comorbidities have been demonstrated. Our results reveal that the repetitive administration of CoPP clearly inhibited the mechanical and thermal allodynia in both hind paws on the fourth day of treatment, and basal values were achieved on day 5 of treatment. GYY4137 administration was also demonstrated to be effective in the treatment of PTX-induced neuropathic pain. Indeed, significant reductions in mechanical and cold allodynia were detected in both hind paws at the third day of treatment; the full reversal of both symptoms was achieved at the fourth day. These results show the painkilling effects of both CoPP and GYY4137 treatments during neuropathic pain caused by PTX. These data are in accordance with the analgesic actions of CoPP and/or CORM-2 (a slow CO releaser) in mice with inflammatory pain or nerve-injury-induced neuropathic pain [37,38], and in animals with vincristine-induced neuropathic pain [11,12]. Our results also agree with the demonstrated analgesic actions of other slow-releasing H_2_S donors, such as isothiocyanates, during osteoarthritis and neuropathic pain [17,18,39], as well as with the antinociceptive effects of GYY4137 in the first stages of PTX-induced neuropathic pain [40] and in animals with CIPN induced by oxaliplatin [16].

This study confirmed the emotional disorders associated with PTX-induced neuropathic pain [24] by demonstrating anxiolytic-like behaviors in the EPM and OF tests and depressive-like behaviors in the FST and TST. Interestingly, both the CoPP and GYY4137 treatments inhibited these affective disorders. That is, both drugs reversed the low number of entrances into the open arms in the EPM test and the low time spent in the central area of the OF test observed in PTX-injected mice treated with vehicle. None of the variables associated with locomotor activity, such as the number of entries into the closed arms of the EPM test or the number of squares crossed in the OF test, were changed, thus suggesting that locomotion alterations did not influence the results. Regarding the depressive-like behaviors associated with PTX-induced neuropathic pain, both the CoPP and GYY4137 treatments normalized the increase in the immobility time in the TST and FST observed in PTX-injected mice, showing the anxiolytic and antidepressant effects induced by the activation of HO-1 and H_2_S during PTX-induced neuropathic pain. These results agree with the antidepressant and anxiolytic effects induced by H_2_S in animals with neuropathic pain associated with diabetes [19] or induced by nerve injury [20].

Our results further revealed the antidepressant effects of CoPP in basal conditions, as demonstrated by the significant reduction in the immobility time observed in vehicle plus CoPP treated mice as compared with vehicle plus vehicle treated animals in the FST and TST. In accordance with our results, other studies have also revealed the antidepressant effects induced by CoPP in the TST [41], as well as with those produced by the adenovirus-inducted overexpression of HO-1 in the FST and TST [42]. Moreover, and supporting our data these antidepressant effects were performed without altering the number of squares crossed in the OF [41,42], thus excluding the possibility that the antidepressant actions induced by HO-1 activation might result from alterations of locomotor activity. The lack of anxiolytic and antidepressant effects induced by GYY4137 under basal conditions has also been previously demonstrated [20]. Nonetheless, our findings revealed, for the first time, the curative potential of CO and H_2_S against the anxiety- and depressive-like behaviors associated with PTX-induced neuropathic pain. Considering the lack of effective treatments with which to alleviate the nociceptive responses and the emotional disorders associated with CIPN, the anxiolytic, antidepressant, and antinociceptive effects of CoPP and GYY4137 in PTX-induced neuropathic pain can help to improve the adverse effects induced by this chemotherapeutic agent.

Regarding the plausible mechanisms underlying these behavioral results, it is well known that the DRG is one of the structures most severely affected by PTX, as well as a pain-processing area that is crucially implicated in CIPN [28]. Accordingly, our results showed that PTX upregulated the expression of p-P38, p-ERK 1/2, PI3K, and p-Akt in the DRG. These results agree with the ones obtained in other work [25], which also show an increase in p-ERK 1/2 and p-P38 expression in the DRG of PTX-injected animals, whose activation increased neuron excitability through the activation of channels such as Nav1.7, facilitating pain sensation. P38 phosphorylation has also been linked to increased NFκB expression and subsequent inflammatory pain in several pain models [25,43]. In accordance with our results, it has been also documented that the PTX-mediated nociceptive symptomatology is also related to the activation of the PI3K/Akt signaling pathway in the DRG, followed by an enhanced expression of inflammatory cytokines [26]. Our data demonstrate the normalization of ERK 1/2 and P38 phosphorylation in GYY4137-treated mice, indicating that, during PIPN, this H_2_S donor mediates part of its analgesic effects by inhibiting ERK 1/2 and P38 activation and, probably, the increased neuronal excitability and inflammatory responses induced by them. By contrast, CoPP treatment did not reduce the activation of either MAPK, meaning that its painkilling properties are probably realized in other ways. Unexpectedly, we did not observe any effect of CoPP or GYY4137 under the overexpression of PI3K and its downstream target p-Akt, a signaling pathway implicated in pain modulation [26,44,45]. This suggests that the antinociceptive effects induced by both compounds are not primarily mediated by inhibiting this signaling pathway, at least at the doses tested in this study.

Considering the involvement of oxidative stress in the development of CIPN, we evaluated the effects of CoPP and GYY4137 on the expression of some oxidative-stress markers and antioxidant enzymes. Our results support the oxidative-stress responses induced by chemotherapeutic agents [28,29] by showing an increased expression of 4-HNE and MDA, two oxidative-stress markers, in the DRG of PTX-injected mice. Interestingly, both CoPP and GYY4137 stabilized the enhanced expression of 4-HNE and MDA, revealing the potent antioxidative actions of both treatments in the PNS of PTX-injected mice. Our data further show an enhancement of the expression of the antioxidant enzymes HO-1 and SOD-1 and the maintenance of the high levels of NQO1 in the DRG of CoPP- and GYY4137-treated mice. This suggests that the antioxidant properties of CoPP and GYY4137 might be implicated in their analgesic effects, as previously demonstrated with the potent analgesic effects induced by several antioxidant agents such as oxindoles and sulforaphane, in animals with nerve-injury-induced neuropathic pain [46,47].

It has been demonstrated that, as a consequence of the multiple alterations caused by PTX, the apoptotic signaling pathways are activated in the PNS [28,32]. An increased expression of BAX was consistently shown in the DRG of mice with PTX-induced neuropathic pain, which was completely reversed by both CoPP and GYY4137, thus demonstrating the anti-apoptotic properties of both treatments in the PNS. These anti-apoptotic properties might also promote the analgesic effects of both compounds during PIPN.

In this study, we also evaluated the effects of CoPP and GYY4137 in the PFC of PTX-injected mice to explore possible pathways implicated in their anxiolytic and antidepressant effects in these animals. PTX induced p-P38, p-ERK 1/2, PI3K, and p-Akt overexpression in this brain area. Although no treatment normalized the high levels of p-Akt and PI3K, CoPP and GYY4137 inhibited the upregulated levels of p-P38 and CoPP further normalized the PTX-induced ERK 1/2 activation. In agreement with our findings, previous studies have demonstrated the inhibitory effects of CoPP and several Nrf2 activators, such as oltipraz, on the expression of p-P38 and p-ERK 1/2 in the spinal cord and/or PFC of animals with nerve-injury-induced neuropathic pain [10,48,49]. Consequently, and taking into account the fact that P38 and ERK 1/2 phosphorylation are implicated in the anxiodepressive-like behaviors by inducing inflammatory responses and oxidative stress in the mouse cortex and that its inhibition reversed the emotional alterations associated with chronic inflammatory pain [50], it is reasonable to propose that the normalization of p-P38 and/or p-ERK 1/2 pathways induced by CoPP and/or GYY4137 in the PFC might be implicated in their anxiolytic and antidepressant effects in animals with PIPN.

PTX was also demonstrated to induce oxidative stress in the PFC by the increased expression of 4-HNE and MDA in this brain area. Moreover, although only CoPP was able to regulate the overexpression of both oxidative-stress markers in the PFC, both treatments upregulated the protein levels of three analyzed antioxidant enzymes (HO-1, NQO1, and SOD-1) in this brain area. Since oxidative stress has been described as a relevant pathological factor contributing to depressive and anxiety disorders [19], the antidepressant and/or anxiolytic effects of CoPP and GYY4137 in animals with PTX-induced neuropathic pain could be mediated by triggering their antioxidant actions as demonstrated by these treatments in other pain models [15].

It is also well known that microglia play a decisive role in the development of anxiodepressive-like behaviors [31]. Accordingly, our results showed microglial activation in the PFC of PTX-injected mice. Moreover, GYY4137 reversed the increased expression of CD11b/c in the PFC, which might explain, at least in part, the antidepressant and/or anxiolytic effects of this H_2_S donor in this pain model.

Finally, we demonstrated that the BAX levels in the PFC were not affected by PTX or by any of the tested treatments, suggesting that, at 21 days after the injection of the antineoplastic drug, the molecular deregulations affecting the PFC did not trigger apoptosis according to the analysis of the BAX levels in this brain area.

## 5. Conclusions

In summary, our results demonstrate that both CoPP and GYY4137 treatments inhibited the neuropathic pain, anxiety- and depressive-like behaviors induced by PTX. Both treatments potentiated the antioxidant responses and/or inhibited oxidative stress in the DRG and/or PFC of mice with PIPN. CoPP and GYY4137 also normalized some plasticity changes in the PFC and/or DRG, and inhibited the apoptotic reactions induced by PTX in the DRG; only GYY4137 inhibited microglial activation in the PFC. In conclusion, this study proposes CoPP and GYY4137 as good candidates for treating PTX-induced neuropathic pain and its accompanying emotional disorders.

## Data Availability

The data presented in this study are available in article.
